# Social Media Use in Interventions for Diabetes: Rapid Evidence-Based Review

**DOI:** 10.2196/10303

**Published:** 2018-08-10

**Authors:** Elia Gabarron, Eirik Årsand, Rolf Wynn

**Affiliations:** ^1^ Norwegian Centre for E-health Research University Hospital of North Norway Tromso Norway; ^2^ Department of Clinical Medicine Faculty of Health Sciences The Arctic University of Norway Tromso Norway; ^3^ Division of Mental Health and Addictions University Hospital of North Norway Tromso Norway

**Keywords:** social media, social networking, health promotion, intervention studies, diabetes

## Abstract

**Background:**

Health authorities recommend educating diabetic patients and their families and initiating measures aimed at improving self-management, promoting a positive behavior change, and reducing the risk of complications. Social media could provide valid channel to intervene in and deliver diabetes education. However, it is not well known whether the use of these channels in such interventions can help improve the patients’ outcomes.

**Objective:**

The objective of our study was to review and describe the current existing evidence on the use of social media in interventions targeting people affected with diabetes.

**Methods:**

A search was conducted across 4 databases (PubMed, Scopus, EMBASE, and Cochrane Library).The quality of the evidence of the included primary studies was graded according to the Grading of Recommendations Assessment, Development and Evaluation criteria, and the risk of bias of systematic reviews was assessed by drawing on AMSTAR (A MeaSurement Tool to Assess systematic Reviews) guidelines. The outcomes reported by these studies were extracted and analyzed.

**Results:**

We included 20 moderate- and high-quality studies in the review: 17 primary studies and 3 systematic reviews. Of the 16 publications evaluating the effect on glycated hemoglobin (HbA_1c_) of the interventions using social media, 13 reported significant reductions in HbA_1c_ values. The 5 studies that measured satisfaction with the interventions using social media found positive effects. We found mixed evidence regarding the effect of interventions using social media on health-related quality of life (2 publications found positive effects and 3 found no differences) and on diabetes knowledge or empowerment (2 studies reported improvements and 2 reported no significant changes).

**Conclusions:**

There is very little good-quality evidence on the use of social media in interventions aimed at helping people with diabetes. However, the use of these channels is mostly linked to benefits on patients’ outcomes. Public health institutions, clinicians, and other stakeholders who aim at improving the knowledge of diabetic patients could consider the use of social media in their interventions.

## Introduction

The prevalence of diabetes has been growing worldwide for the last few decades [1], and it has become one of the four priority noncommunicable diseases targeted by world leaders, together with cardiovascular disease, cancer, and chronic respiratory disease [1]. All types of diabetes can lead to complications, reduce the quality of life, and increase the risk of premature death [1,2]. To support clinical practice, health authorities recommend educating diabetic patients and their families and initiating prevention measures aimed at improving self-management and promoting a positive behavior change, thereby reducing the risk of complications [1-7].

The use of social media has increased dramatically in the recent years [8], and social media channels could be effective in supporting clinical practice and delivering education to improve self-management and to promote a positive behavior change among people affected with chronic diseases [9]. However, it is not well known whether the use of these channels in interventions can help improve diabetic patients’ outcomes, and the evidence of using social media in interventions for people with diabetes needs to be updated.

Evidence on positive effects of social media interventions on health behavior-related outcomes (ie, weight loss and physical activity) exists in 2 meta-analyses focusing on several health conditions [10,11]. However, 2 other meta-analyses have reported mixed results regarding the use of social media in health interventions [12,13]. Furthermore, a third meta-analysis concluded that using social media did not contribute to reducing risk factors in patients with noncommunicable diseases [14].

Norway is one of the most connected countries in the world, and most of the Norwegian population uses social media [15,16]. Due to its ubiquity, a health promotion intervention using social media, aimed at people affected by diabetes and their relatives, is now being initiated by our research team [17]. An updated status on the evidence that exists regarding the use and usefulness of social media in diabetes is essential. Hence, the objective of this paper was to review and describe the current evidence on the use of social media in interventions targeting people with diabetes.

## Methods

### A Rapid Review

We performed a rapid review to quickly capture the current evidence on the use of social media in interventions on diabetes. We had two research questions: (1) Is there evidence on the use of social media in interventions aimed at improving, maintaining, or promoting health among people affected with diabetes? and (2) What are the reported outcomes, for example, the effects on clinical parameters, effects on behavior, or other effects?

The rapid review method was chosen as it typically provides similar conclusions as systematic reviews, and it allows to quickly and efficiently access the current evidence on the topic [18-21]. In this rapid review, we followed the Preferred Reporting Items for Systematic Reviews and Meta-Analyses (PRISMA) [22] and the MeaSurement Tool to Assess systematic Reviews (AMSTAR) [23,24] guidelines. This review has been registered in PROSPERO (registration number: CRD42018088206).

### Search Strategy

To answer the research questions, we performed an electronic search on February 13, 2018. It covered published studies comprising the terms “Social media,” “Social networking,” “Facebook,” “Twitter,” or “YouTube” in combination with the term “Diabetes” included in the title or abstract and indexed in the following databases: PubMed (Medical Subject Heading terms and text word), Scopus, EMBASE, and the Cochrane Library. The search strategy was limited to studies published in English. The full search strategy is summarized in Multimedia Appendix 1.

### Inclusion and Exclusion Criteria

Publications were included in the review if they (1) focused on diabetes or involved participants affected by diabetes; (2) described interventions aimed at improving, maintaining, or promoting health; (3) reported results from the intervention; and (4) used social media in the intervention. Both primary studies and reviews were considered to be of interest and were, therefore, included in this review. Papers that did not meet all four criteria were excluded from the review.

### Eligibility and Data Extraction

All references captured by the search engine were uploaded to EndNote X7 (Clarivate Analytics; Philadelphia, PA, USA). Duplicates were identified and removed. To assess the eligibility of the papers, two passes were done. In the first pass, all titles and abstracts were examined by one reviewer (EG). In the second pass, the full text of the studies selected on the first pass was extracted and carefully analyzed to confirm their eligibility. When it was unclear whether the studies should be included, they were discussed and agreed with a second reviewer (EÅ). The agreed upon studies were included in the quality assessment. A single reviewer (EG) extracted the data from the included studies. The following data were extracted: interventions (duration and participants), social media use (channels, use as main tool for the intervention or as supporting tool), and outcomes (effects on clinical parameters, on behavior, or other effects).

### Quality Evidence Assessment and Risk of Bias

The quality of evidence and risk of bias of the studies included in this review were classified by one reviewer (EG). The quality of evidence of primary studies was assessed following the Grading of Recommendations Assessment, Development and Evaluation guidelines [25]. A second reviewer (RW) verified the assigned quality of a random sample of primary studies. The risk of systematic bias was assessed by drawing on the AMSTAR criteria [19,23,24].

## Results

### Sample

**Figure 1:**
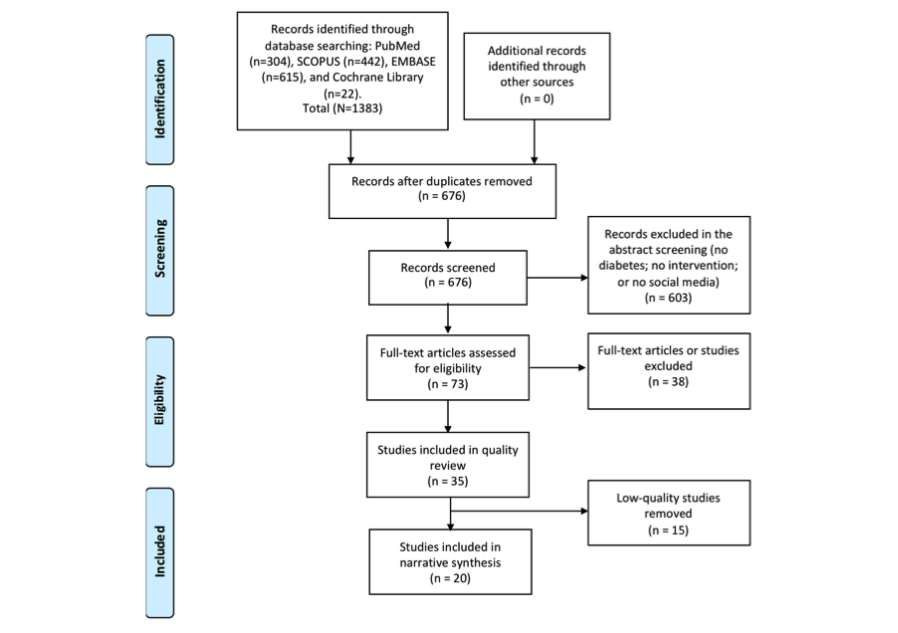
Preferred Reporting Items for Systematic Reviews and Meta-Analyses (PRISMA) flowchart of the selection procedure.

A total of 1383 publications were identified, and after removing duplicates, 676 titles and abstracts were screened. The full search strategy and its results are summarized in Multimedia Appendix 1. The list of all potentially relevant studies that were read in full-text form but were excluded from the review can be found in Multimedia Appendix 2. Of these publications, 35 met the inclusion criteria [26-60]; of them, 32 were primary studies [26-32,34-38,40-59] and 3 were systematic reviews [33,39,60] (Figure 1).

### Quality of The Evidence and Risk of Bias Assessment

Only 1 of the 35 included studies was considered to be of high quality [31]; 19 studies were considered to be of moderate quality: the 3 systematic reviews [33,39,60,61] (Multimedia Appendix 3), all 15 randomized controlled trials (RCTs) [26-30,32,34,35,42,44,45,48,54,55,58], and 1 nonrandomized intervention [40]. The remaining 15 nonrandomized studies were weighted as being of low or very low quality, and therefore, they were removed from the narrative synthesis (these studies are listed in Multimedia Appendix 4). Hence, 20 studies (1 of high quality and 19 of moderate quality) were included in this review. Multimedia Appendix 5 summarizes these 20 studies.

The PRISMA checklist of this study can be found in Multimedia Appendix 6.

#### Evidence: Clinical Effects

Of the 20 included studies, 19 reported clinical outcomes and only 1 study did not refer to any clinical effect [55].

##### Glycated Hemoglobin

The main reported clinical outcome was glycated hemoglobin (HbA_1c_). Eighteen publications evaluated HbA_1c_ values and reported heterogeneous effects. On one hand, 4 publications reported significant improvements in HbA_1c_ values, favoring the groups that used social media comparison with control groups. This effect has been reported in 2 systematic reviews summarizing the evidence from studies focusing on both type one diabetes (T1D) and type two diabetes (T2D) and in 2 RCTs focusing specifically on T1D [27,33,39,44]. In one of these systematic reviews two analyses were performed: one of them was a meta-analysis including RCTs only; in the second analysis, nonrandomized studies were also included. In the latter, a significant mean reduction in HbA_1c_ values was found, favoring the social media group (0.49%, 95% CI −0.64 to −0.34, I^2^=86%) [39]. On the other hand, 13 RCTs reported significant improvements in HbA_1c_ values among all the study participants, independently of whether they were allocated to the group where social media was used or not. These studies mostly targeted young people affected with T1D [26,28,30,32,34,35,45,54,60]. Furthermore, another study reported mixed results [58]. The data analysis of all the participants included in this study (208 adults with T2D) did not show significant decreases in HbA_1c_ values. However, in a second analysis, it was found that patients with HbA_1c_≥10% at baseline had a significant decrease at 6 months [58]. Two additional studies focusing on young people with T1D did not find any differences in HbA_1c_ values [31,42].

##### Blood Pressure

Effects on blood pressure were reported only by 2 systematic reviews, both finding improvements associated with social media use. One of the systematic reviews referred to 5 studies with a total of 2580 patients (1317 in the intervention groups and 1263 in the control groups), where there was a significant mean difference in systolic blood pressure (3.47 mmHg, 95% CI 5.01 to 1.94, *P*<.001, I^2^=0%) and diastolic blood pressure (1.84 mmHg, 95% CI 2.98 to 0.70, *P*=.002, I^2^=29%), favoring the intervention groups using social media [39]. The second review also found reductions in blood pressure associated with Web 2.0 participation, reported in 2 studies [33].

##### Other Reported Clinical Outcomes

Additional evidence on clinical effects has been reported for triglycerides and severe hypoglycemia episodes. Only 1 systematic review referred to the effect on triglycerides. This review reported the effect drawing on 10 studies with a total of 989 patients. A significant reduction of 11.05% (95% CI: 20.92 to 1.18, *P*=.03, I^2^=0) was found among the study participants where social media had been used [39]. An RCT carrying out a 1-year intervention did not find any differences in severe hypoglycemia episodes for any of the study participants [31].

#### Evidence: Effects on Behavior

Of the 20 included studies, 16 referred to different effects on behavior.

##### Satisfaction With the Intervention

Five publications had comparable findings related to patients’ high satisfaction with the interventions where social media were used or to internet visits being preferred by patients [26,28,30,39,60].

##### Health-Related Quality of Life

Five studies reported on this item, reaching different conclusions. Two studies, an RCT and a systematic review, reported improvements in health-related quality of life (HRQoL) among T1D and T2D patients linked to social media use [33,44]. Three RCTs with young T1D patients did not find any differences regarding HRQoL between the groups after the intervention [27,31,48].

##### Diabetes Knowledge and Diabetes Empowerment

This effect was reported in 4 studies and with mixed conclusions. One study involving adolescents with T1D found significantly increased diabetes knowledge on treatment or testing in the intervention group at 4, 8, and 12 months [42]. In another study on young people with T1D, it was found that social media use allowed patients to gain knowledge and information about diabetes and interact when making their daily insulin adjustments [45]. On the other hand, 2 studies, both referring to a 1-year intervention carried out in Sweden with T1D patients, showed no improvement in diabetes empowerment [27,31].

##### Medication Adherence

Two publications reported on treatment adherence. No differences were found regarding adherence in a systematic review [33], while the social media intervention group of an RCT had significantly better medication adherence (*P*=.01) [29].

##### Healthier Life-Styles

There were mixed results on this outcome as well. A systematic review described reductions in dietary fat intake, but the same review also found no effects on physical activity [33]. However, a recently published RCT found a significant improvement in self-reported physical activity (est 0.28, *P*=.046) for those with the highest engagement in the site and a decreased sedentary time (Fitbit data) for the intervention group compared with that for a control group (est −12.17, *P*=.048) [58].

##### Other Reported Behavioral Outcomes

Additional reported effects on behavior were an increase in diabetes communication (*P*<.05) and medical self-efficacy (*P*<.01) [48], reductions in depressive symptoms [33], and no significant differences in perceived quality of care [31]. Table 1 summarizes the evidence identified in this review on clinical and behavioral outcomes of interventions using social media and addressing people with diabetes.

#### Targeted Population

Most (14/20) of the publications focused only on young people affected with T1D, with different age groups ranging from 0 to 23 years (as specified in the studies) [26-32,34,35,42,44,45,48,54]. Five publications referred to both T1D and T2D patients, and therefore, participants had a broader age range [33,39,40,55,60]. Only one study specifically targeted adults diagnosed with T2D [58]. The included primary studies were performed in Macedonia [26,28,30,32,34,35,44,45,54], Sweden [27,31], the United States [48,55], and Ireland [42], and the location was unspecified in 3 of the publications [29,40,58].

#### Social Media Use

The main use of social media was as a supporting tool for the main intervention (14/20), primarily used for reinforcing regular visits and with the purpose of engaging patients in treatment and improving self-management and diabetes control. In these cases, the chosen social media channels were Facebook (group), Facebook (chat), Skype, specific social networking sites, or social media in general [26-28,30-32,34,35,40,44,45,48,55,58]. Of these studies, 7 (all of them belonging to the same research group) reported improvements in HbA_1c_ values for all study participants [26,28,30,32,34,35,45]. Two studies found improvements in HbA_1c_ values only in the participants allocated to the social media groups [27,44]. Two studies did not find any differences in HbA_1c_ values when social media was used as a supporting tool [31,48]. Moreover, one study found mixed results, with no improvements in HbA_1c_ values for the whole sample but improvements for the participants in whom the values were higher at baseline [58]. Two publications did not report on HbA_1c_ values linked to the use of social media as a supporting tool [40,55].

**Table 1:** Summary of the evidence on reported outcomes (n=20).

Outcomes		Supported by number of publications			
		Significant positive effects	Mixed results	No significant differences	Outcome not reported
**Clinical effects**					
	HbA_1c_^b^	13^a^ [26-28,30,32-35,39,44, 45,54,60]	1 [58]	4 [29,31,42,48]	2 [40,55]
	Blood pressure	2 [33,39]	0	0	18 [26-32,34,35,40,42,44,45,48, 54,55,58,60]
	Triglycerides	1 [39]	0	0	19 [26-35,40,42,44,45,48, 54,55,58,60]
	Severe hypoglycemia	0	0	1 [31]	19 [26-30,32-35,39,40,42,44,45,48, 54,55,58,60]
**Effects on behavior**					
	HRQoL^c^	2 [33,44]	0	3 [27,31,48]	15 [26,28-30,32,34,35,39,40,42,44, 54,55,58,60]
	Knowledge or empowerment	2 [42,45]	0	2 [27,31]	16 [26,28-30,32-35,39,40,44,48,54, 55,58,60]
	Medication adherence	1 [29]	0	1 [33]	18 [26-28,30-32,34,35,39,40,42,44, 45,48,54,55,58,60]
	Healthier self-reported life-styles	1 [58]	1 [33]	0	18 [26-32,34,35,39,40,42,44,45,48, 54,55,60]
	Self-efficacy	1 [40]	1 [48]	0	18 [26-35,39,42,44,45,54, 55,58,60]
	Depressive symptoms	1 [33]	0	0	19 [26-32,34,35,39,40,42,44,45, 48,54,55,58,60]
	Perceived quality of care	0	0	1 [31]	19 [26-30,32-35,39,40,42,44,45,48, 54,55,58,60]

^a^13 studies reported improvements in HbA_1c_ values in all study participants; 4 of these studies reported improvements only in the intervention groups (comparison with control groups) [27,33,39,44].

^b^HbA_1c_: Glycated hemoglobin.

^c^HRQoL: health-related quality of life.

Three RCTs studied social media as the main channel for delivering the intervention. These 3 studies used peers in educational and behavioral interventions aimed at young people affected with T1D. The purpose of these interventions was to increase diabetes knowledge and to improve clinical outcomes. These 3 studies used Facebook closed groups and Viber [29,42,54]. Two of them did not find any significant differences in HbA_1c_ values [29,42], while the third study reported improvements in HbA_1c_ values among all the participants [54]. The use of social media as a main channel for delivering the intervention or as a supporting tool was not clearly stated in any of the 3 systematic reviews [33,39,60].

## Discussion

### Summary of the Evidence

A rapid review method was used to quickly capture the current evidence on the use of social media in interventions on diabetes. Following a search in 4 databases, only 20 publications considered of adequate quality were included in this review: 3 systematic reviews and 17 primary studies.

The research evidence shows that the most commonly reported outcome in intervention studies using social media is HbA_1c_, followed by satisfaction with the intervention, HRQoL, and diabetes knowledge or empowerment. Most of the intervention studies using social media that evaluated HbA_1c_ values reported significant improvements (13/16 publications) [26-28,30,32-35,39,44,45,54,60]. Four of these publications, 2 systematic reviews and 2 RCTs, reported improvements only in intervention groups compared with that in control groups [27,33,39,44]. However, due to a heterogeneity in the methods that were used in the studies, including differences in the characteristics of participants, sample sizes, and study lengths, comparing them is difficult.

The 5 studies that measured satisfaction with the interventions where social media were used unanimously reported positive effects [26,28,30,39,60]. Two publications reported positive effects on HRQoL [33,44], and 2 others found improvements in knowledge [42,45], while 3 publications did not report any significant differences in HRQoL [27,31,48] or any improvements in knowledge [27,31].

### Should We Use Social Media in Interventions for People With Diabetes?

Health authorities have recommended educating diabetic patients and their families with the aim of improving self-management, promoting a positive behavior change, and reducing the risk of complications [1-7]. Although the use of social media has not been linked to clear improvements in one meta-analysis focusing on patients with noncommunicable diseases [14], there are several other meta-analyses where some favorable effects have been found among people affected with chronic diseases [10-13,39]. The findings of the present review suggest that the use of social media in interventions for diabetes in many cases has been beneficial, and we did not find any studies that suggested worsened outcomes with this type of intervention. Studies using social media in their interventions have mostly showed superior results linked to the use of social media. Only one of the publications included in this review did not report any benefit on clinical or behavioral outcomes [30]. In this case, the researchers used social media in a 1-year intervention, and they used their own social media channel [31].

It is interesting to note that more than half of the studies used social media as a tool or resource to enhance the main intervention, and in these cases, the interventions resulted in improvements in HbA_1c_ values. Participants in these studies who were allocated to receive education either through Facebook chat or Skype as reinforcement of the main intervention had significant decreases in HbA_1c_ values [26,28,30,32,34,35,45]. Furthermore, compared with patients in the control group, improvements in HbA_1c_ levels were found among patients receiving health education through closed groups on Facebook and were found in one study using its own social media network [27,44]. In only 2 studies where social media was used as a supporting tool no differences in HbA_1c_ values were found; these 2 studies used their own social media channel [31,48].On the other hand, only 1 of the 3 studies that used social media as a main channel for delivering the intervention and measured HbA_1c_ values [29,42,54] reported significant improvements. This study used Viber communication for delivering doctor or peer support [54].

These findings suggest that using social media as a supporting tool for the main intervention is beneficial for improving health outcomes in T1D patients. Furthermore, it seems that the clinical improvement is most likely to happen when the chosen social media is one of the most popular networking sites.

Our review has mainly identified studies conducted with young T1D populations; however, our conclusions are comparable to those reported in a meta-analysis published in 2014 that predominantly analyzed studies involving T2D patients [39]. Therefore, we think that public health institutions, clinicians, and other stakeholders should consider the use of social media in their interventions targeting people affected with diabetes. However, special attention should be paid to the risk of misinformation or harmful health material that can coexist when carrying out interventions in open social media channels as it could lead to undesirable or unexpected effects [62-67].

### Knowledge Gaps and Next Directions

Most of the included studies focused only on young people affected with T1D, probably because it is believed that these media are typically used by young people. Certainly, since the origin of social media, younger people have been the most frequent users of these channels. However, the presence of older generations on social media has increased in recent years, and about 80% of North Americans and Norwegians in their 40s report being social media users [8,68]. Hence, including older populations through social media would also make sense. This could be an especially valuable way of targeting people affected with T2D, a disease that is mostly diagnosed in adulthood and whose prevalence has dramatically increased in the last few decades [1]. Intervention studies using social media seem to improve health outcomes in T1D patients, and they could be beneficial for people with T2D as well. However, more research, using social media, on diabetes types is needed to answer this question.

In this review, we identified many abstracts presented at conferences, but there were fewer full papers reporting methods and results in detail. Knowing further details of the method used and the interventions could help identify the mechanisms or behavior techniques that work better for improving patient outcomes. So far, it seems that studies that use social media as supporting tool and where the social media is used for delivering health education report better outcomes. However, there are not enough studies where social media was used as the main channel for delivering the intervention. In future research, one should consider using different social media channels as main sources for delivering the intervention.

In research projects, it is more common to use restricted-access social media (ie, Facebook closed groups, Facebook chat, Skype, etc), which allows the researcher to have a better control of the environment and the contents and also protect the patients’ privacy. However, the use of open social media channels offers the possibility of a large-scale impact. Providing high-quality contents on diabetes through the most commonly used open social media channels and interacting with the social media users could potentially help people with diabetes. By having access to free-of-charge quality information, they could improve their knowledge, an important prerequisite for improving self-management and health behaviors. Further research should explore how to best use open social media channels for health promotion interventions in diabetes.

### Strengths and Limitations

Our results and conclusions might be susceptible to bias as a consequence of streamlining the systematic review process. There might be a selection bias (failure to search in additional potentially relevant databases, only 1 reviewer selecting the studies) and a publication bias (we only searched in 4 databases; we did not search for gray literature; and our search was limited to the English language). Eight of the included studies conducted in Macedonia could be based on the same study, although we treated the reported results independently, as they provided different sample sizes, different age ranges of the included participants, and different intervention periods. Because many of the included publications were abstracts presented at conferences and because we did not have access to complete data, a quantitative synthesis was not possible.

### Conclusion

There is little evidence on the use of social media in interventions aimed at people affected with diabetes. However, after weighing the existing evidence, it seems that the use of these channels is predominantly linked to beneficial patient outcomes. Public health institutions, clinicians, and other stakeholders who aim at improving diabetes patient education should consider the use of social media in their interventions.

## Appendix

Multimedia Appendix 1 Search strategy (search date: February 13, 2018).

Multimedia Appendix 2 List of all potentially relevant studies that were read in full-text form but excluded from the review.

Multimedia Appendix 3 Risk of bias assessment of the systematic reviews included in the review according to the MeaSurement Tool to Assess systematic Reviews (AMSTAR) criteria (n=3).

Multimedia Appendix 4 List of excluded studies because of low Grading of Recommendations Assessment, Development and Evaluation (GRADE) scores.

Multimedia Appendix 5 Summary of publications included in the review (n=20).

Multimedia Appendix 6 Preferred Reporting Items for Systematic Reviews and Meta-Analyses (PRISMA) checklist [22].

## Abbreviations

HbA_1c_: glycated hemoglobin
HRQoL: health-related quality of life
RCT: randomized controlled trial
T1D: type 1 diabetes
T2D: type 2 diabetes

## References

[1] (2016). World Health Organization.

[2] (2012). International Diabetes Federation.

[3] Lange K, Swift P, Pańkowska E, et al (2014). ISPAD Clinical Practice Consensus Guidelines 2014. Diabetes education in children and adolescents. Pediatr Diabetes.

[4] Montgomery B (2018). The Pillars of Prevention: Discover, Advocate, and Educate. Diabetes Spectr.

[5] National Clinical Guideline Centre (UK) (2015). Type 1 Diabetes in Adults: Diagnosis and Management. London: National Institute for Health and Care Excellence (UK); 2015 Aug. National Institute for Health and Care Excellence: Clinical Guidelines.

[6] National Collaborating Centre for Women's and Children's Health (UK) (2015). Diabetes (Type 1 and Type 2) in Children and Young People Diagnosis and Management. National Collaborating Centre for Women's and Children's Health (UK). London: National Institute for Health and Care Excellence (UK); 2015 Aug.

[7] Schaper NC, Van NJJ, Apelqvist J, et al (2016). Prevention and management of foot problems in diabetes: a Summary Guidance for Daily Practice 2015, based on the IWGDF Guidance Documents. Diabetes Metab Res Rev.

[8] (2018). Pew Research Center.

[9] Coiera E (2013). Social networks, social media, and social diseases. BMJ.

[10] Laranjo L, Arguel A, Neves AL, et al (2015). The influence of social networking sites on health behavior change: a systematic review and meta-analysis. J Am Med Inform Assoc.

[11] An R, Ji M, Zhang S (2017). Effectiveness of Social Media-based Interventions on Weight-related Behaviors and Body Weight Status: Review and Meta-analysis. Am J Health Behav.

[12] Välimäki M, Athanasopoulou C, Lahti M, Adams CE (2016). Effectiveness of Social Media Interventions for People With Schizophrenia: A Systematic Review and Meta-Analysis. J Med Internet Res.

[13] Williams G, Hamm MP, Shulhan J, et al (2014). Social media interventions for diet and exercise behaviours: a systematic review and meta-analysis of randomised controlled trials. BMJ Open.

[14] Mita G, Ni MC, Jull A (2016). Effectiveness of social media in reducing risk factors for noncommunicable diseases: a systematic review and meta-analysis of randomized controlled trials. Nutr Rev.

[15] (2018). Translate Media.

[16] (2018). Statista.

[17] Gabarron E, Bradway M, Fernandez-Luque L, et al (2018). Social media for health promotion in diabetes: study protocol for a participatory public health intervention design. BMC Health Serv Res.

[18] Garritty C, Stevens A, Gartlehner G, et al (2016). Cochrane Rapid Reviews Methods Group to play a leading role in guiding the production of informed high-quality, timely research evidence syntheses. Syst Rev.

[19] Kelly SE, Moher D, Clifford TJ (2016). Quality of conduct and reporting in rapid reviews: an exploration of compliance with PRISMA and AMSTAR guidelines. Syst Rev.

[20] Khangura S, Konnyu K, Cushman R, et al (2012). Evidence summaries: the evolution of a rapid review approach. Syst Rev.

[21] Tricco AC, Antony J, Zarin W, et al (2015). A scoping review of rapid review methods. BMC Med.

[22] Moher D, Liberati A, Tetzlaff J, et al (2009). Preferred reporting items for systematic reviews and meta-analyses: the PRISMA statement. J Clin Epidemiol.

[23] Shea BJ, Hamel C, Wells GA, et al (2009). AMSTAR is a reliable and valid measurement tool to assess the methodological quality of systematic reviews. J Clin Epidemiol.

[24] Shea B, Reeves Bc, Wells G, et al (2017). AMSTAR 2: a critical appraisal tool for systematic reviews that include randomised or non-randomised studies of healthcare interventions, or both. BMJ.

[25] Meerpohl JJ, Langer G, Perleth M, et al (2012). [GRADE guidelines: 3. Rating the quality of evidence (confidence in the estimates of effect)]. Z Evid Fortbild Qual Gesundhwes.

[26] Petrovski G, Zivkovic M, Stratrova SS, et al (2010). Carelink, Skype and Facebook improve diabetes control in adolescents on pump therapy. Diabetologia.

[27] Hanberger L, Ludvigsson J, Nordfeldt S (2013). Use of a web 2.0 portal to improve education and communication in young patients with families: randomized controlled trial. J Med Internet Res.

[28] Petrovski G, Dimirovski C, Bogoev M, et al (2011). Internet visits improve diabetes control in adolescents on pump therapy. Diabetes Technology & Therapeutics.

[29] Lapp J, White NH (2012). Social networking and peer support (SNAPS) in adolescents with type 1 diabetes mellitus (T1D): A pilot study. Diabetes.

[30] Petrovski G, Milenkovic T, Petrovska I, et al (2012). Social media and diabetes: can we improve glucose control in adolescents on pump therapy? One year experience. Diabetes.

[31] Hanberger L, Ludvigsson J, Nordfeldt S (2013). Use of a web 2.0 portal to improve education and communication in young patients with families: randomized controlled trial. J Med Internet Res.

[32] Petrovski G, Milenkovic T, Jovanovska B, et al (2013). Can we improve glucose control in type 1 diabetics on insulin pump using social media and diabetes ? One year experience. Diabetes Technol Ther.

[33] Stellefson M, Chaney B, Barry AE, et al (2013). Web 2.0 chronic disease self-management for older adults: a systematic review. J Med Internet Res.

[34] Petrovski G, Milenkovic MZT, Jovanovska B, et al (2014). One-year experience of using social media as tool to improve glucose control in adolescents with type 1 diabetes : a crossover study. Diabetes.

[35] Petrovski G, Milenkovic T, Subeska S, et al (2014). Social media and diabetes: a tool to improve glucose control in type 1 diabetic adolescents on insulin pump: cross-over study. Diabetes Technol Ther.

[36] Saboo B, Chandarana HK, Talaviya P, et al (2014). Impact of use of social media in patients with type 1 diabetes for management of diabetes. Pediatric Diabetes.

[37] Scaramuzza A, Bosetti A, Redaelli F, et al (2014). To whatsapp or not to whatsapp? What could be done with new social media to manage type 1 diabetes in adolescents. Diabetes Technology & Therapeutics.

[38] Thomas J, Donaldson BJL (2014). Sugar Free with Justin T.: Diabetes education through community partnerships. Journal of Extension.

[39] Toma T, Athanasiou T, Harling L, et al (2014). Online social networking services in the management of patients with diabetes mellitus: systematic review and meta-analysis of randomised controlled trials. Diabetes Res Clin Pract.

[40] Gómez-Zúñiga B, Pousada M, Hernandez MM, et al (2015). The Online Big Blue Test for Promoting Exercise: Health, Self-Efficacy, and Social Support. Telemed J E Health.

[41] Lim PK, Cheng TS, Hui YCA, et al (2015). D-buddy peer support for better health outcomes in adolescents with diabetes mellitus. International Journal of Pediatric Endocrinology.

[42] McDarby V, Hevey D, Cody D (2015). Evaluation of a social media site to improve diabetes glycaemic control, knowledge and self-efficacy: the ASSIST study. Pediatr Diabetes.

[43] Ng SM (2015). Improving patient outcomes with technology and social media in paediatric diabetes. BMJ Qual Improv Rep.

[44] Petrovski G, Zivkovic M (2015). Facebook as a useful tool to improve glucose control in patients with type 1 diabetes: One year follow-up study. Diabetes Technology & Therapeutics.

[45] Petrovski G, Zivkovic M, Stratrova SS (2015). Social media and diabetes: can Facebook and Skype improve glucose control in patients with type 1 diabetes on pump therapy? One-year experience. Diabetes Care.

[46] Rothenberg R, Zetelski M, Sivitz J, et al (2015). Use of smartphone, a cellular glucometer and social media app in the management of type 1 DM in the adolescent population: The future of diabetes care. Horm Res Paediatr.

[47] Spehar UA, Bogdanic A, Krnic N, et al (2015). Diabetes-management empowerment intervention 'Youth for adolescents with type 1 diabetes'. Pediatric Diabetes.

[48] Whittemore R, Jeon S, Liberti L, et al (2015). Implementation and efficacy of two internet interventions for teens with T1D. Diabetes.

[49] Wilson L, Allardice B, Brillante N, et al (2015). The role of My Diabetes My Way (MDMW) social media sites in promoting diabetes education and self-management. Diabet. Med.

[50] Yi-Frazier JP, Cochrane K, Mitrovich C, et al (2015). Using Instagram as a Modified Application of Photovoice for Storytelling and Sharing in Adolescents With Type 1 Diabetes. Qual Health Res.

[51] Yi-Frazier JP, Mitrovich C, Pascual M, et al (2015). Using instagram to improve outcomes in adolescents with type 1 diabetes (T1D): A feasibility study. Diabetes.

[52] Blackstock S, Solomon S, Watson M, et al (2016). The use of a WhatsAppTM broadcast group to improve knowledge and engagement of adolescents with type 1 diabetes. Archives of Disease in Childhood.

[53] Marsland N, Twenefour D, Kelly T (2016). Impact of 'Enjoy Food': Diabetes UK's programme to promote healthy eating. Diabetic Medicine.

[54] Petrovski G, Zivkovik M, Ahmeti I, et al (2016). Mobile social media and diabetes: Viber communication improves glucose control in type 1 diabetes patients on insulin pump. Diabetes Technology and Therapeutics.

[55] Pyatak E, Carandang K, Diaz J, et al (2016). Resilient, empowered, active living (real) with diabetes: Implementing an occupational therapy (OT) intervention for diabetes management. Diabetes.

[56] Kariyawasam D, Pender S, Jones M, et al (2017). An evaluation of a novel programme for empowering young people with Type 1 diabetes: YES -Youth Empowerment Skills. Diabetic Medicine.

[57] Marsland N, Twenefour D, Elvin E (2017). Impact of 'Enjoy Food': Diabetes UK's programme to promote healthy eating. Diabetic Medicine.

[58] Vorderstrasse A, Melkus GD, Feinglos M, et al (2017). Virtual environment for diabetes self-management education and support: Preliminary RCT outcomes. Circulation. Conference: Resuscitation Science Symposium, ReSS.

[59] Wilson L, Cunningham SG, Allardice B, et al (2017). My diabetes my way: Delivering innovative diabetes care. Diabetic Medicine.

[60] Alanzi T (2018). Role of Social Media in Diabetes Management in the Middle East Region: Systematic Review. J Med Internet Res.

[61] Seo H, Kim KU (2012). Quality assessment of systematic reviews or meta-analyses of nursing interventions conducted by Korean reviewers. BMC Med Res Methodol.

[62] Gabarron E, Serrano JA, Wynn R, Lau AYS (2014). Tweet content related to sexually transmitted diseases: no joking matter. J Med Internet Res.

[63] Lau AYS, Gabarron E, Fernandez-Luque L, Armayones M (2012). Social media in health--what are the safety concerns for health consumers?. Health Inf Manag.

[64] Oyeyemi SO, Gabarron E, Wynn R (2014). Ebola, Twitter, and misinformation: a dangerous combination?. BMJ.

[65] Pal BR (2014). Social media for diabetes health education - inclusive or exclusive?. Curr Diabetes Rev.

[66] Weitzman ER, Kelemen S, Garvey KC (2013). Social networking for care improvement and panel management. Diabetes.

[67] Wynn R, Oyeyemi SO, Johnsen J-A, et al (2017). Tweets are not always supportive of patients with mental disorders. International J Integrated Care.

[68] (2018). Statista.

