# Supplementary material for: Social Media Use in Interventions for Diabetes: Rapid Evidence-Based Review
Source: J Med Internet Res. 2018 Aug 10;20(8):e10303. doi: 10.2196/10303 (PMC6109225; doi:10.2196/10303)
Supplement: Multimedia Appendix 1 [file jmir_v20i8e10303_app1.pdf]

DATABASE	SEARCH ENGINE	OUTPUT
PubMed	Social media[Title/Abstract] AND Diabetes[Title/Abstract]	132
PubMed	Social networking[Title/Abstract] AND Diabetes[Title/Abstract]	48
PubMed	Facebook[Title/Abstract] AND Diabetes[Title/Abstract]	36
PubMed	Twitter[Title/Abstract] AND Diabetes[Title/Abstract]	25
PubMed	YouTube[Title/Abstract] AND Diabetes[Title/Abstract]	9
PubMed	"Social Media"[Mesh] AND "Diabetes Mellitus"[Mesh]	54
PubMed	"Social Media"[Mesh] AND "Diabetes Insipidus"[Mesh]	0
PubMed	"Social Media"[Mesh] AND "Diabetes, Gestational"[Mesh]	0
Scopus	TITLE-ABS-KEY ("Social media") AND TITLE-ABS-KEY (*diabetes) AND (LIMIT-TO [DOCTYPE, "ar"] OR LIMIT-TO [DOCTYPE, "cp"]) AND (LIMIT-TO [LANGUAGE, "English"]) AND (LIMIT-TO [SRCTYPE, "j"] OR LIMIT-TO ([SRCTYPE, "p"])	195
Scopus	TITLE-ABS-KEY ("Social networking") AND TITLE-ABS-KEY (*diabetes) AND (LIMIT-TO [SRCTYPE, "j"] OR LIMIT-TO [SRCTYPE, "p"]) AND (LIMIT-TO [DOCTYPE, "ar"] OR LIMIT-TO [DOCTYPE, "cp"]) AND (LIMIT-TO [LANGUAGE, "English"])	146
Scopus	TITLE-ABS-KEY ("Facebook") AND TITLE-ABS-KEY (*diabetes) AND (LIMIT-TO [SRCTYPE, "j"] OR LIMIT-TO [SRCTYPE, "p"]) AND (LIMIT-TO [DOCTYPE, "ar"] OR LIMIT-TO [DOCTYPE, "cp"]) AND (LIMIT-TO [LANGUAGE, "English"])	45
Scopus	TITLE-ABS-KEY ("Twitter") AND TITLE-ABS-KEY (*diabetes) AND (LIMIT-TO [SRCTYPE, "j"] OR LIMIT-TO [SRCTYPE, "p"]) AND (LIMIT-TO [DOCTYPE, "ar"] OR LIMIT-TO [DOCTYPE, "cp"]) AND (LIMIT-TO [LANGUAGE, "English"])	43
Scopus	TITLE-ABS-KEY ("Yotube") AND TITLE-ABS-KEY (*diabetes) AND (LIMIT-TO [SRCTYPE, "j"] OR LIMIT-TO [SRCTYPE, "p"]) AND (LIMIT-TO [DOCTYPE, "ar "] OR LIMIT-TO [DOCTYPE, "cp"]) AND (LIMIT-TO [LANGUAGE, "English"])	13

EMBASE	([exp *social media].ti,ab,kw. AND exp *diabetes).ti,ab,kw. Limit to (English language)	362
EMBASE	([exp *social networking].ti,ab,kw. AND exp *diabetes).ti,ab,kw. Limit to (English language)	77
EMBASE	([exp *Facebook].ti,ab,kw. AND exp *diabetes).ti,ab,kw. Limit to (English language)	103
EMBASE	([exp *Twitter].ti,ab,kw. AND exp *diabetes).ti,ab,kw. Limit to (English language)	63
EMBASE	([exp *YoutTube].ti,ab,kw. AND exp *diabetes).ti,ab,kw. Limit to (English language)	10
Cochrane Library	"'Social media' AND 'Diabetes' in Title, Abstract, Keywords in Other Reviews"	5
Cochrane Library	"'Social networking' AND 'Diabetes' in Title, Abstract, Keywords in Other Reviews"	7
Cochrane Library	"'Facebook' AND 'Diabetes' in Title, Abstract, Keywords in Other Reviews"	5
Cochrane Library	"'Twitter' AND 'Diabetes' in Title, Abstract, Keywords in Other Reviews"	3
Cochrane Library	"'YouTube' AND 'Diabetes' in Title, Abstract, Keywords in Other Reviews"	2
