# Supplementary material for: Social Media Use in Interventions for Diabetes: Rapid Evidence-Based Review
Source: J Med Internet Res. 2018 Aug 10;20(8):e10303. doi: 10.2196/10303 (PMC6109225; doi:10.2196/10303)
Supplement: Multimedia Appendix 2 [file jmir_v20i8e10303_app2.pdf]

- Tsai CC, Tsai SH, Zeng-Treitler Q, Liang BA. Patient-centered consumer health social network websites: a pilot study of quality of user-generated health information. AMIA Annual Symposium proceedings AMIA Symposium. 2007:1137.
- Mieres JH, Phillips LM. The interdisciplinary approach to culturally tailored medical care: "Social networking" for decreasing risk: Comment on "The effects of a nurse case manager and a community health worker team on diabetic control, emergency department visits, and hospitalizations among urban African Americans with type 2 diabetes mellitus: a randomized controlled trial" and "Trial of family and friend support for weight loss in African American adults". Archives of internal medicine. 2009;169(19):1804-5.
- Sprod R, Agostinho S, Harper B, editors. What spaces? Designing authentic, sustainable online learning spaces for children with diabetes. ASCILITE 2009 - The Australasian Society for Computers in Learning in Tertiary Education; 2009.
- Pemu PE, Quarshie AQ, Josiah-Willock R, Ojutalayo FO, Alema-Mensah E, Ofili EO. Socio-demographic psychosocial and clinical characteristics of participants in e-HealthyStrides(c): an interactive ehealth program to improve diabetes self-management skills. Journal of health care for the poor and underserved. 2011;22(4 Suppl):146-64.
- Weitzman ER, Adida B, Kelemen S, Mandl KD. Sharing data for public health research by members of an international online diabetes social network. PloS one. 2011;6(4):e19256.
- Balfe M, Doyle F, Conroy R. Using Facebook to recruit young adults for qualitative research projects: how difficult is it? Computers, informatics, nursing : CIN. 2012;30(10):511-5.
- Fain JA. Is the diabetes educator better off now compared to last year? The Diabetes educator. 2012;38(6):759.
- Lin YH, Chen RR, Guo SH, Chang HY, Chang HK. Developing a web 2.0 diabetes care support system with evaluation from care provider perspectives. Journal of medical systems. 2012;36(4):2085-95.
- McPherson AC, Price K. Does an interactive website provide additional support to young people participating in an educational intervention for type 1 diabetes ? Pediatric diabetes. 2012;13:139.
- Vissenberg C, Nierkens V, Uitewaal PJM, Geraci D, Middelkoop BJC, Nijpels G, et al. The DISC (Diabetes in Social Context) Study-evaluation of a culturally sensitive social network intervention for diabetic patients in lower socioeconomic groups: A study protocol. BMC Public Health. 2012;12(1).
- Collins SE, Lewis DM. Social media made easy: Guiding patients to credible online health information and engagement resources. Clinical Diabetes. 2013;31(3):137-41.
- Barnes NA, Asante-Bediako E. Diabetes youth care-support group for young people living with diabetes mellitus in Ghana. Pediatric diabetes. 2014;19):104-5.
- Gray KM, Clarke K, Alzougool B, Hines C, Tidhar G, Frukhtman F. Internet protocol television for personalized home-based health

information: design-based research on a diabetes education system. JMIR research protocols. 2014;3(1):e13.

- Kim HH, Seo HJ. HealthTWITTER Initiative: Design of a Social Networking Service Based Tailored Application for Diabetes Self-Management. Healthcare informatics research. 2014;20(3):226-30.
- Lee R, Whitley HP. Use of social media to support patients with diabetes mellitus. The Consultant pharmacist : the journal of the American Society of Consultant Pharmacists. 2014;29(1):53-7.
- Valdez RS, Guterbock TM, Thompson MJ, Reilly JD, Menefee HK, Bennici MS, et al. Beyond traditional advertisements: leveraging Facebook's social structures for research recruitment. Journal of medical Internet research. 2014;16(10):e243.
- Cole E. Diabetes care tool puts kids in control. Nursing standard (Royal College of Nursing (Great Britain) : 1987). 2015;29(45):18-20.
- Gomez-Galvez P, Suarez Mejias C, Fernandez-Luque L. Social media for empowering people with diabetes: Current status and future trends. Conference proceedings: Annual International Conference of the IEEE Engineering in Medicine and Biology Society IEEE Engineering in Medicine and Biology Society Annual Conference. 2015;2015:2135-8.
- Hernandez M. Diabetes social media: a tool to engage patients. Canadian journal of diabetes. 2015;39(3):194.
- Hilliard ME, Sparling KM, Hitchcock J, Oser TK, Hood KK. The emerging diabetes online community. Current diabetes reviews. 2015;11(4):261-72.
- Kraaij V, Garnefski N. Cognitive, behavioral and goal adjustment coping and depressive symptoms in young people with diabetes: a search for intervention targets for coping skills training. Journal of clinical psychology in medical settings. 2015;22(1):45-53.
- McDarby V, Hevey D, Cody D. An overview of the role of social network sites in the treatment of adolescent diabetes. Diabetes technology & therapeutics. 2015;17(4):291-4.
- Ramadas A, Chan CK, Oldenburg B, Hussien Z, Quek KF. A web-based dietary intervention for people with type 2 diabetes: development, implementation, and evaluation. International journal of behavioral medicine. 2015;22(3):365-73.
- Sherr D, Lipman RD. The Diabetes Educator and the Diabetes Self-management Education Engagement: The 2015 National Practice Survey. The Diabetes educator. 2015;41(5):616-24.
- Alanzi T, Istepanian R, Philip N. Design and Usability Evaluation of Social Mobile Diabetes Management System in the Gulf Region. JMIR research protocols. 2016;5(3):e93.
- Boudreau F, Moreau M, Cote J. Effectiveness of Computer Tailoring Versus Peer Support Web-Based Interventions in Promoting Physical Activity Among Insufficiently Active Canadian Adults With Type 2 Diabetes: Protocol for a Randomized Controlled Trial. JMIR research protocols. 2016;5(1):e20.

- Colorafi K. Connected health: a review of the literature. mHealth. 2016;2:13.
- George KS, Roberts CB, Beasley S, Fox M, Rashied-Henry K. Our Health Is in Our Hands: A Social Marketing Campaign to Combat Obesity and Diabetes. American journal of health promotion : AJHP. 2016;30(4):283-6.
- Hudnut-Beumler J, Po'e E, Barkin S. The Use of Social Media for Health Promotion in Hispanic Populations: A Scoping Systematic Review. JMIR public health and surveillance. 2016;2(2):e32.
- Kim HS, Jeong YJ, Baik SJ, Yang SJ, Kim TM, Kim H, et al. Social Networking Services-Based Communicative Care for Patients with Diabetes Mellitus in Korea. Applied clinical informatics. 2016;7(3):899-911.
- Kurashvili R, Gabritchidze S, Shelestova E, Tsutskiridze L, Zhulina A. Project diabetes prevention and care improvement realized by welfare foundation georgia in collaboration with georgian union of diabetes and endocrine associations. Endocrine Practice. 2016;22 (Supplement 6):3-4.
- Lewinski AA, Fisher EB. Social interaction in type 2 diabetes computer-mediated environments: How inherent features of the channels influence peer-to-peer interaction. Chronic illness. 2016;12(2):116-44.
- Li J, Kong J, editors. Cell phone-based diabetes self-management and social networking system for American Indians. 2016 IEEE 18th International Conference on e-Health Networking, Applications and Services, Healthcom 2016; 2016.
- Patrakeeva E, Zagorovskaya T. Webinar as an alternative format of psychological help to people with diabetes. Diabetes Technology and Therapeutics. 2016;1):A96.
- Ruddock JS, Poindexter M, Gary-Webb TL, Walker EA, Davis NJ. Innovative strategies to improve diabetes outcomes in disadvantaged populations. Diabetic medicine : a journal of the British Diabetic Association. 2016;33(6):723-33.
- Han P, Nicholson W, Norton A, Singrman R, Sundaresan A, Bennett WL, editors. Development and preliminary findings of diabetes sisters voices-an online community to engage women with diabetes about research and healthcare priorities. 40th annual meeting of the society of general internal medicine, SGIM 2017; 2017; United States: Journal of General Internal Medicine.
- Rogers EA, Fine SC, Handley MA, Davis HB, Kass J, Schillinger D. Engaging Minority Youth in Diabetes Prevention Efforts Through a Participatory, Spoken-Word Social Marketing Campaign. American journal of health promotion : AJHP. 2017;31(4):336-9.
- Zoppis I, Mauri G, Sicurello F, Santoro E, Castelnuovo G, editors. DIABESITY: Design of mHealth integrated solutions for empowering diabetic and obese citizens in self-monitoring and self-management using mobile devices, apps, social media and web-based technologies. Communication, Management and Information Technology - Proceedings of the International Conference on Communication, Management and Information Technology, ICCMIT 2016; 2017.
