# Supplementary material for: Social Media Use in Interventions for Diabetes: Rapid Evidence-Based Review
Source: J Med Internet Res. 2018 Aug 10;20(8):e10303. doi: 10.2196/10303 (PMC6109225; doi:10.2196/10303)
Supplement: Multimedia Appendix 3 [file jmir_v20i8e10303_app3.pdf]

Criteria	Stellfson, 2013	Toma, 2014	Alanzi, 2018
Was an "a priori" design provided?	No	No	No
Was there duplicate study selection and data extraction?	Yes[a]	Yes	No
Was a comprehensive literature search performed?	Yes	No	No
Was the status of publication (ie, gray literature) used as an inclusion criterion?	No	No	Yes
Was a list of studies (included and excluded) provided?	No	No	Yes
Were the characteristics of the included studies provided?	Yes	No	Yes
Was the scientific quality of the included studies assessed and documented?	Yes	Yes	No
Was the scientific quality of the included studies used appropriately in formulating conclusions?	Yes	Yes	No
Were the methods used to combine the findings of studies appropriate?	Yes	Yes	Yes
Was the likelihood of publication bias assessed?	Yes	Yes	No
Was the conflict of interest included?	Yes	Yes	Yes
Total points	8 Moderate quality[b]	6 Moderate quality	5 Moderate quality

[a]Each item scoring ''yes'' is awarded with 1 point. The points are added, with 11 as the maximum score [19,23,68].
[b]Low-quality AMSTAR scores ranged from 0 to 4, moderate quality from 5 to 8, and high quality from 9 to 11 [68].
