# Supplementary material for: Social Media Use in Interventions for Diabetes: Rapid Evidence-Based Review
Source: J Med Internet Res. 2018 Aug 10;20(8):e10303. doi: 10.2196/10303 (PMC6109225; doi:10.2196/10303)
Supplement: Multimedia Appendix 4 [file jmir_v20i8e10303_app4.pdf]

- Lim PK, Cheng TS, Hui YCA, Lim STJ, Lek N, Yap F, et al. D-buddy peer support for better health outcomes in adolescents with diabetes mellitus. International Journal of Pediatric Endocrinology Conference: 8th Biennial Scientific Meeting of the Asia Pacific Paediatric Endocrine Society, APPES. 2015((Suppl 1)):P15.
- Spehar Uroic A, Bogdanic A, Krnic N, Rojnic Putarek N, Grubic M. Diabetes-management empowerment intervention "youth for adolescents with type 1 diabetes". Pediatric diabetes. 2015;21):136-7.
- Kariyawasam D, Pender S, Jones M, Mullings O, Jackson P, Antebi T, et al. An evaluation of a novel programme for empowering young people with Type 1 diabetes: YES -Youth Empowerment Skills. Diabetic Medicine. 2017;34 (Supplement 1):121.
- Marsland N, Twenefour D, Elvin E. Impact of 'Enjoy Food': Diabetes UK's programme to promote healthy eating. Diabetic Medicine. 2017;34 (Supplement 1):128.
- Marsland N, Twenefour D, Kelly T. Impact of 'Enjoy Food': Diabetes UK's programme to promote healthy eating. Diabetic Medicine. 2016;1):117.
- Saboo B, Chandarana HK, Talaviya P, Sarvaiya PK, Shah SJ, Hasnani DJ, et al. Impact of use of social media in patients with type 1 diabetes for management of diabetes. Pediatric diabetes. 2014;19):67.
- Ng SM. Improving patient outcomes with technology and social media in paediatric diabetes. BMJ quality improvement reports. 2015;4(1).
- Wilson L, Cunningham SG, Allardice B, Wake DJ. My diabetes my way: Delivering innovative diabetes care. Diabetic Medicine. 2017;34 (Supplement 1):116-7.
- Wilson L, Allardice B, Brillante M, Cunningham SG, Elmsie-Smith A, McAlpine RR, et al. The role of My Diabetes My Way (MDMW) social media sites in promoting diabetes education and self-management. Diabetic Medicine. 2015;1):134-5.
- Thomas JB, Donaldson JL. Sugar Free with Justin T.: Diabetes education through community partnerships. Journal of Extension. 2014;52(6).
- Scaramuzza A, Bosetti A, Redaelli F, Gazzarri A, Rossi E, Ferrari M, et al. To whatsapp or not to whatsapp? what could be done with new social media to manage type 1 diabetes in adolescents. Diabetes Technology and Therapeutics. 2014;1):A160-A1.
- Blackstock S, Solomon S, Watson M, Kumar P. The use of a WhatsAppTM broadcast group to improve knowledge and engagement of adolescents with type 1 diabetes. Archives of Disease in Childhood. 2016;101 (Supplement 1):A315-A6.
- Rothenberg R, Zetelski M, Sivitz J, Klein G, Chartoff A, Pearson JA, et al. Use of smartphone, a cellular glucometer and social media app in the management of type 1 DM in the adolescent population: The future of diabetes care. Hormone Research in Paediatrics. 2015;1):374-5.

- Yi-Frazier JP, Cochrane K, Mitrovich C, Pascual M, Buscaino E, Eaton L, et al. Using Instagram as a Modified Application of Photovoice for Storytelling and Sharing in Adolescents With Type 1 Diabetes. Qualitative health research. 2015;25(10):1372-82.
- Yi-Frazier JP, Mitrovich C, Pascual M, Cochrane K, Beauregard N, Velasco K, et al. Using instagram to improve outcomes in adolescents with type 1 diabetes (T1D): A feasibility study. Diabetes. 2015;1):A671.
