# Supplementary material for: Social Media Use in Interventions for Diabetes: Rapid Evidence-Based Review
Source: J Med Internet Res. 2018 Aug 10;20(8):e10303. doi: 10.2196/10303 (PMC6109225; doi:10.2196/10303)
Supplement: Multimedia Appendix 5 [file jmir_v20i8e10303_app5.pdf]

| Reference | Study type | Intervention duration | Participants | Social media use (Channel) | Outcomes |
|---|---|---|---|---|---|
| Stellfson, 2013 [33] | Systematic review | Several durations | 8 studies specifically on diabetes | Not specified<br><br>(Social media in general) | Clinical effects<br>A 4-month study period, Web 2.0 engagement among individuals with diabetes was not associated with any improvements in biological or clinical outcomes.<br>2 RCT[a]s reported greater declines in HbA1c[b] associated to Web 2.0 participation, and 2 RCTs reported reductions in blood pressure.<br><br>Effects on behavior<br>Medication adherence: 2 RCTs studying individuals with T2D[c] suggested that self-monitoring using Web 2.0 did not improve medication adherence.<br>Physical activity: 1 RCT found no effect of Web 2.0 participation on physical activity. In other studies of individuals with diabetes, self-monitoring of physical activity behaviors improved with concomitant reductions in dietary fat intake.<br>Health-related quality of life and depressive symptoms: several RCTs of individuals with diabetes reported that Web 2.0 participation was associated with improved generic health-related quality of life and a reduction in depressive symptoms. |
| Toma, 2014 [39] | Systematic review | Several durations | 34 RCTs | Not specified<br><br>(Social media in general) | Clinical effects<br>HbA1c: significant mean difference of 0.45% (95% CI: 0.60 to 0.29, $P$<0.001) favoring intervention groups (social media) between baseline and follow-up but with significant heterogeneity ($I^2$=81%). Meta-analysis including non-randomized additional studies showed a significant mean reduction in HbA1c favoring the intervention groups (0.49%, 95% CI: −0.64 to −0.34, $I^2$=86%).<br>Triglycerides: at follow-up: 10 studies with a total of 989 patients reported significant reduction of 11.05% (95% CI: 20.92 to 1.18, $P$=0.03, $I^2$=0) in those using social media.<br>Systolic and diastolic blood pressure: 5 studies with a total of 2580 patients (1317 intervention and 1263 control) reported significant mean difference in systolic (3.47 mmHg, 95% CI: 5.01to 1.94, $P$<0.001, $I^2$ = 0%) and diastolic (1.84 mmHg, 95% CI: 2.98 to 0.70, $P$=0.002, $I^2$=29%) blood pressures on follow-up, favoring the intervention groups. |
| Alanzi, 2018 [60] | Systematic review | Several durations | 5 studies | Not specified<br><br>(Social media in general) | Clinical effects<br>Significant decrease in HbA1c values reported in 1 study. |
| Nordfeldt, 2011 [27] | Randomized intervention | 1 year | 463 participants (young people with T1D[d] and their families) | As supporting tool of the intervention<br><br>(Own social networking in the portal) | Clinical effects<br>Those patients where someone in the family visited the website 5 times or more had lower HbA1c after 1 year ($P$=0.01).<br><br>Effects on behavior<br>No effects were found on health-related quality of life, empowerment, or quality of information. |

| | | | | | |
|---|---|---|---|---|---|
| Petrovski, 2010 [26] | Randomized intervention | 6 months | 38 patients with T1D using insulin pumps with glucose sensors (ages 13-22) | As supporting tool of the intervention<br><br>(Facebook chat and Skype) | Clinical effects<br>Significant HbA1c improvement in both groups (groups 1 and 2: 7.4±0.9% and 7.5±1.1% on beginning and 6.2±0.8% and 6.3±1.0%, *P*<0.05, at 6 months, respectively).<br><br>Effects on behavior<br>Internet visits were more preferred by patients. |
| Petrovski, 2011 [28] | Randomized intervention | 6 months | 42 patients with T1D using insulin pumps with glucose sensors (ages 14-23) | As supporting tool of the intervention<br><br>(Facebook chat and Skype) | Clinical effects<br>Significant HbA1c improvements in both intervention and control groups (groups 1 and 2: 7.6 ±0.9% and 7.7±1.1% on beginning, and 6.3±0.8% and 6.2±1.0%, *P*<0.05, at the end of the study, respectively).<br><br>Effects on behavior<br>Internet visits were more preferred by patients. |
| Petrovski, 2012 [30] | Randomized intervention | 1 year | 78 patients with T1D using insulin pumps with glucose sensors (ages 14-23) | As supporting tool of the intervention<br><br>(Facebook chat and Skype) | Clinical effects<br>HbA1c improvements in both groups (*P*<0.05).<br>HbA1c in Group 1 went from 7.45±0.9% to 6.22±0.8% and from 7.68±1.1% to 6.09±1.0% in Group 2 at 1 year. The significant improvement was performed in the first 6 months and it was maintained.<br><br>Effects on behavior<br>The authors state that internet visits were preferred by patients, but no numbers or methods measuring this were reported. |
| Petrovski, 2013 [32] | Randomized intervention | 1 year | 91 adolescents with T1D (ages 12-21) | As supporting tool of the intervention<br><br>(Facebook chat and Skype) | Clinical effects<br>HbA1c improvements in both groups (*P*<0.05). HbA1c in Group 1 went from 7.65±0.9% to 6.12±0.8% at 12 months and from 7.59±1.1% to 6.05±1.0% in Group 2. |
| Petrovski, 2014a [34] | Randomized intervention | 1 year | 124 adolescents with T1D (ages 16-23) | As supporting tool of the intervention<br><br>(Facebook chat and Skype) | Clinical effects<br>HbA1c improvements in both groups (*P*<0.05). HbA1c in Group 1 went from 7.8±0.7% to 6.4±0.8%, and from 7.9±1.0% to 6.3±1.2% in Group 2 at 6 months. After crossover, patients maintained HbA1c values. |
| Petrovski, 2014b [35] | Randomized intervention | 1 year | 114 adolescents with T1D (ages 13-23) | As supporting tool of the intervention<br><br>(Facebook chat and Skype) | Clinical effects<br>HbA1c improvements in both groups (*P*<0.05). HbA1c in Group 1 went from 7.8±0.7% to 6.4±0.8% and from 7.9±1.0% to 6.3±1.2% in Group 2 at 6 months. After crossover, patients maintained HbA1c values (6.5±0.7% and 6.4±0.6%). |
| Petrovski, 2015a [45] | Randomized intervention | 1 year | 56 patients with T1D | As supporting tool of the | Clinical effects<br>Both groups improved HbA1c at 12 months. Regular group: 7.7±1.6% (61±17.5 mmol/mol) at |

| | | | | | |
|---|---|---|---|---|---|
| | | | using insulin pumps with glucose sensors (ages 14-23) | intervention<br><br>(Facebook chat and Skype) | baseline versus 6.6±1.5% (49±16.4 mmol/mol) at 12 months. Improvement occurred in the first 6 months and was maintained for 6 additional months.<br><br>Effects on behavior<br>The authors found that social media use allowed the patients to gain diabetes knowledge and information and interact in their daily insulin adjustments. |
| Petrovski, 2015b [44] | Randomized intervention | 1 year | 441 patients with T1D | As supporting tool of the intervention<br><br>(Facebook group) | Clinical effects<br>Significant decrease of 0.7% in HbA1c was found in intervention group (7.9±1.2% vs. 7.2±0.8%, $P<0.05$) compared to 0.2% in control group (7.9±1.4% vs. 7.6±1.9%, $P=0.72$).<br><br>Effects on behavior<br>Diabetes Quality of Life for Youth Inventory questionnaire showed a significant improvement ($P<0.05$) only in patients from intervention group. |
| Petrovski, 2016 [54] | Randomized intervention | 6 months | 72 T1D patients on insulin pumps | As main source for delivering the intervention<br><br>(Viber) | Clinical effects<br>Significant decrease of HbA1c (7.8±1.6% to 7.1±1.2% in Viber+Doctor+Educator group and 7.7±1.1% to 7.2±1.9% in Viber+Patients group). |
| Hanberger, 2013 [31] | Randomized intervention | 1 year | 163 T1D patients (ages 0-18) and their families | As supporting tool of the intervention<br><br>(Own social networking in the portal) | Clinical effects<br>No differences were found at baseline and after study year 1 between the intervention and control group, adolescents and parents, respectively, regarding the outcome variables (HbA1c, severe hypoglycemia, frequency of blood glucose self-control).<br><br>Effects on behavior<br>No differences were found at baseline and after study year 1 between the intervention and control group, adolescents and parents, respectively, regarding the outcome variables (HRQoL[e], empowerment, and perception of quality of care regarding information measured by DISABKIDS, Swe-DES-SF-10[f], and QPP[g] scales, respectively). |
| Whittemore, 2015 [48] | Randomized intervention | 6 months | 124 teens with T1D (ages 11-14) | As supporting tool of the intervention<br><br>(Own social media) | Clinical effects<br>Teens in both programs had no changes in HbA1c over 6 months.<br><br>Effects on behavior<br>There was no significant difference in self-management, perceived stress, diabetes self-efficacy, and quality of life between groups. Teens in both groups had an increase in diabetes communication ($P<0.05$) and medical self-efficacy ($P<0.01$). Posts to the discussion forum were higher in the social media group. |
| Vorderstrasse, 2017 [58] | Randomized intervention | 6 months | 208 adults with T2D (mean age 59) | As supporting tool of the intervention<br><br>(Own social media) | Clinical effects<br>HbA1c did not decrease significantly overall However, among those with HbA1c >10% at baseline there was a significant decrease (mean 11.42±1.53 to 8.77±1.49, $P=0.018$).<br><br>Effects on behavior<br>Significant improvement in self-reported physical activity (est 0.28, $P=0.046$) for those with |

| | | | | | |
|---|---|---|---|---|---|
| | | | | | the highest engagement in the site, and decreased sedentary time (Fitbit data) for the intervention group compared to control group (est −12.17, *P*=0.048). |
| Lapp, 2012 [29] | Randomized intervention | 3 months | 46 adolescents with T1D (mean age in intervention group 14.6, and in control group 15.4) | As main source for delivering the intervention<br><br>(Social media channel not specified) | Clinical effects<br>HbA1c did not statistically change between baseline and 3 months (9.6±1.5% at baseline vs. 9.8±1.5% at 3 months in intervention group and 10.1±1.8% at baseline vs. 9.9±2.0% at 3 months in control group)<br><br>Effects on behavior<br>Trend (*P*=0.06) for subjects in intervention group to report reduction in conflict on Diabetes Family Conflict Scale between baseline and 3 months.<br>Parents of children in intervention group reported on PedsQL-DM[h] scale that the children had fewer treatment barriers (*P*=0.015) and better medication adherence (*P*=0.012). |
| McDarby, 2015 [42] | Randomized intervention | 3-month intervention or assessments over 1 year | 73 adolescents with T1D | As main source for delivering the intervention<br><br>(Facebook closed group) | Clinical effects<br>No significant differences in HbA1c.<br><br>Effects on behavior<br>Significantly increased diabetes knowledge on treatment and testing in the intervention group at 4 months, differences maintained at 8 and 12 months. |
| Pyatak, 2016 [55] | Randomized intervention | 6 months | 81 diabetic patients (mean age 22.6) | As supporting tool of the intervention<br><br>(Social media in general) | Effects on behavior<br>Fidelity maintained in over 95% of sessions. |
| Gomez-Zuniga, 2015 [40] | Nonrandomized intervention | Not reported | 3916 people with diabetes | As supporting tool of the intervention<br><br>(Various online social networks) | Clinical effects<br>A mixed factorial analysis of variance comparing insulin-dependents versus noninsulin-dependents at both times of glucose measurement (before and after exercise) indicated that the change in glucose level was more pronounced among insulin-dependent participants, *P*<0.001.<br><br>Effects on behavior<br>The participants who reduced their blood glucose after exercise the least were those with lower self-efficacy (*P*=0.021 and *P*=0.029) and with lower perceived social support (not significant). |

[a]HbA1c: glycated hemoglobin.

[b]RCT: randomized controlled trial.

[c]T2D: Type 2 diabetes.

[d]T1D: Type 1 diabetes.

[e]HRQoL: Health-Related Quality of Life.

[f]Swe-DES-SF-10: Swedish Diabetes Empowerment Scale-short form 10.

[g]QPP: Quality from the Patients' Perspective questionnaire.

[h]PedsQL: Pediatric Quality of Life Inventory.
